# The pathophysiology of glucose intolerance in newly diagnosed, untreated T2DM

**DOI:** 10.1007/s00592-021-01785-9

**Published:** 2021-09-24

**Authors:** Gareth J. Dunseath, Stephen D. Luzio, Rajesh Peter, David R. Owens

**Affiliations:** Diabetes Research Group, Grove Building, Singleton Park, Swansea, SA2 8PP UK

**Keywords:** Glucose intolerance, Type 2 diabetes mellitus, Beta-cell function, Insulin sensitivity

## Abstract

**Aims:**

The two predominant pathophysiological defects resulting in glucose intolerance are beta-cell dysfunction and insulin insensitivity. This study aimed to re-examine beta-cell function and insulin sensitivity across a continuum from normal glucose tolerance (NGT) to early type 2 diabetes (T2DM) employing highly specific insulin, C-peptide and intact proinsulin assays.

**Materials and methods:**

A total of 104 persons with NGT, 85 with impaired glucose tolerance (IGT) and 554 with newly diagnosed T2DM were investigated. Following an overnight fast, all underwent a 4-h standardised mixed meal tolerance test (MTT), and on a second day, a sub-group underwent a frequently sampled insulin-modified intravenous glucose tolerance test (FSIVGTT) over a 3-h period. The participants were stratified according to fasting glucose and BMI for analysis.

**Results:**

The MTT revealed that increasing FPG was accompanied by progressively elevated and delayed postprandial glucose peaks. In parallel, following an initial compensatory increase in fasting and postprandial insulin responses there followed a progressive demise in overall beta-cell secretory capacity. FSIVGTT demonstrated a major reduction in the early insulin response to IV glucose in persons with IGT accompanied by a dramatic fall in insulin sensitivity. Beyond pre-diabetes, ever-increasing fasting and postprandial hyperglycaemia resulted predominantly from a progressively decreasing beta-cell secretory function.

**Conclusion:**

This study utilising improved assay technology re-affirms that beta-cell dysfunction is evident throughout the spectrum of glucose intolerance, whereas the predominant fall in insulin sensitivity occurs early in its evolution.

**Supplementary Information:**

The online version contains supplementary material available at 10.1007/s00592-021-01785-9.

## Introduction

Glucose homeostasis is regulated by a feedback loop, involving beta-cell secretion and insulin sensitivity to maintain glucose concentrations within a tight range [1, 2]. Glucose intolerance, in its initial stages with fasting hyperglycaemia, results from a combination of beta-cell insufficiency in the presence of overwhelming insulin resistance [3, 4]. Further deterioration in glucose homeostasis occurs despite a transitory period when there is a compensatory but delayed increase in overall insulin secretion. Thereafter, there is a progressive demise of beta-cell secretion in the presence of reduced insulin sensitivity [5]. The prevailing and essential defect throughout the natural history of glucose intolerance is dysfunctional beta-cell secretory function, which becomes an ever greater contributor with increasing glycaemia.

Historically, it has been shown that in the early stages in the development of glucose intolerance, fasting hyperglycaemia (impaired fasting glucose) is predominantly due to insulin resistance [6, 7]. However, defects in beta-cell function, assessed by the response to an intravenous glucose bolus, are already present in people with impaired glucose tolerance (IGT) [3].

Many of the pathophysiological studies that highlighted the roles of beta-cell dysfunction and insulin resistance in the development of glucose intolerance were conducted 20 or more years ago [3, 8–16]. These pioneering studies used non-specific radioimmunoassays to define beta-cell secretory capacity to different nutrient challenge tests and methods for defining beta-cell function and insulin sensitivity.

More recent studies [5, 17, 18] describing the relative roles of beta-cell dysfunction and insulin resistance continue to present similar findings, with insulin resistance evident at low levels of hyperglycaemia and beta-cell dysfunction more predominant at higher glycaemic levels. Nevertheless, limitations remain even in these studies, including the inclusion of participants at differing stages of T2DM, continued use of sub-optimal assays lacking specificity, use of different and short-term carbohydrate challenges with limited sampling time points, lack of proinsulin measurement and use of fasting tests to estimate beta-cell function and insulin resistance.

Most recently, there has been a shift in focus towards recognising sub-groups of individuals with glucose intolerance ranging from pre-diabetes to overt type 2 diabetes with their respective risk of developing complications, based on their beta-cell function and insulin sensitivity [19–21].

Therefore, there is a need to revisit this area utilising the recently available more specific immunoassays and standardised challenge tests to re-affirm or modify the historical findings. The data presented here describe the glycaemic and beta-cell secretory responses to two different challenge tests: a standardised mixed meal tolerance test (MTT) and a frequently sampled insulin-modified intravenous glucose tolerance test (FSIVGTT). These were performed within a few days of each other, in people with normal glucose tolerance (NGT), IGT and a large cohort of newly diagnosed, treatment naïve participants with T2DM, not exposed to lifestyle change or pharmacological intervention. Glucose, insulin, C-peptide and intact proinsulin profiles were derived, and indices of beta-cell function, including the ‘gold-standard’ disposition index, and insulin sensitivity are described. The participants were also stratified according to their FPG and BMI to view the findings in the context of increasing glycaemia and obesity.

## Research design and methods

### Study population

Data collection was carried out between 1981 and 2007. A total of 743 participants were recruited within 2 weeks of diagnosis: 104 with NGT, 85 with IGT and 554 newly diagnosed with T2DM, all confirmed by a 2-h 75-g oral glucose tolerance test. Those with T2DM were referred directly from primary care with minimal advice and no pharmacological intervention given. All were confirmed GAD autoantibody negative.

Ethical approval was received from South Glamorgan/Bro Taf Research Ethics Committee, and all participants provided informed consent. Procedures were conducted in accordance with the principles of the Declaration of Helsinki (1996) and Good Clinical Practice.

### Carbohydrate challenge tests

**Fig. 1:**
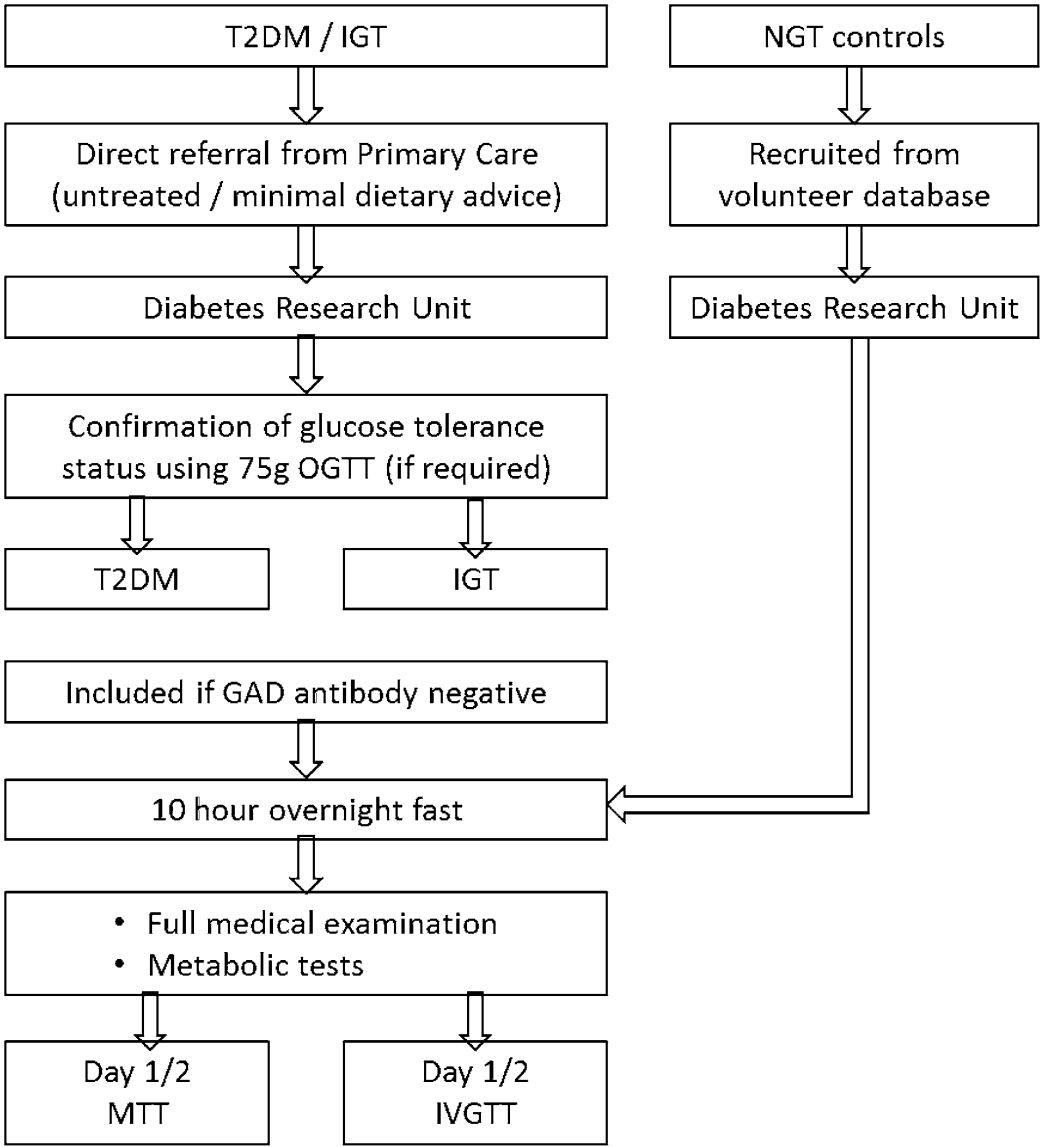
Summary of study procedures

Following a 10-h overnight fast, all subjects underwent a standardised MTT. A FSIVGTT was also performed in a sub-set of 286 subjects (Fig. 1). The carbohydrate challenge tests have previously been described by Albarrak et al. [22], with further details included in Supplementary Tables 1 and 2. All procedures were performed in the Diabetes Research Unit, University Hospital of Wales, and subsequently Llandough University Hospital, Cardiff, UK.

### MTT

The MTT consisted of a solid mixed meal of approximately 500 kcal (calorie contribution: 58% carbohydrate, 22% fat and 20% protein). All subjects had an intravenous cannula inserted for blood sampling, kept patent with a slow-running saline infusion. Baseline blood samples were taken (−30 and −15 min) before the meal was given (0 min) which was consumed within 10 min. Further samples were taken at regular intervals over the 4-h postprandial period (Supplementary Table 2) for determination of plasma glucose, insulin, C-peptide and intact proinsulin.

### FSIVGTT

Baseline fasting samples were taken (−30, −15 and 0 min), before 300 mg/kg body mass of 50% dextrose was administered intravenously over 2 min. At the 20-min time point, an intravenous infusion of insulin was given (Actrapid; 0.03 IU/kg body mass for NGT and 0.05 IU/kg body mass for IGT and T2DM) [22]. Blood samples were taken at frequent intervals during the 3.5-h study period (Supplementary Table 2) for determination of plasma glucose and insulin. The sampling procedure was similar to the MTT; however, an additional cannula was inserted into the contra-lateral arm to deliver the exogenous glucose and insulin.

### Blood collection

Blood was collected into tubes containing fluoride oxalate for determination of glucose and into lithium heparin for insulin, C-peptide and intact proinsulin, separated in a refrigerated centrifuge and the plasma decanted into labelled tubes and stored at −20 °C until assay.

### Laboratory measurements

Glucose was determined using a glucose oxidase method (YSI 2300 Stat Plus, YSI, UK). The insulin, C-peptide and intact proinsulin samples were measured using highly specific immunoassays. The earliest samples were measured as described by Sobey et al. [23]. The assays, using the same antibodies and displaying comparable cross-reactivity and assay characteristics, were subsequently converted to chemiluminescent assays (IV2-001,002 and 004; Invitron Ltd., UK). Cross-reactivity with associated beta-cell products was ≤ 2.0% in all assays.

### Calculated parameters

Maximum concentration (C_max_) was the peak postprandial concentration and time to maximum (T_max_), the time (in minutes) to reach peak. Areas under the response curves (AUC) were calculated using the trapezoidal rule. Insulin/glucose ratio (representing the insulin response relative to the ambient glucose level) was calculated by dividing insulin by the corresponding glucose value (pmol/mmol). Acute insulin response (AIR) was the insulin C_max_ during the first 10 min post-glucose bolus during the FSIVGTT.

### Derived indices

#### Beta-cell function

Postprandial beta-cell responsiveness (M_1_), an indicator of beta-cell function, was derived by modelling the glucose and C-peptide levels before and during the MTT as described by Hovorka et al. [24]. Disposition index was calculated as the product of insulin sensitivity and the indicator of beta-cell function [25].

#### Insulin sensitivity

Insulin sensitivity (SI) was estimated using the minimal model method [26–28], utilising the IS CIBA software [29].

### Statistical analysis

Normality was assessed using the Shapiro–Wilk test and QQ plots. Normally distributed data are displayed as the mean (standard deviation) and compared using t tests. Non-normally distributed data presented as the median (interquartile range) and compared using Kruskal–Wallis with Bonferroni adjusted post hoc analysis.

Participants were divided into sub-groups according to glycaemic status. The IGT group contained both those with isolated IGT and IFG/IGT; as subsequently no differences in beta-cell function or insulin sensitivity were observed between these groups, the groups were combined for analysis. Further sub-division into tertiles of FPG within the T2DM group or BMI within the IGT and T2DM sub-groups was performed to ensure similar numbers could be included for analysis in each comparator group.

- **1) Glucose Tolerance**
  - i) **NGT**
  - ii) IGT
  - iii) T2DM-GT1 (FPG <8.5 mmol/L)
  - iv) T2DM-GT2 (FPG 8.5–11.6 mmol/L) and
  - v) T2DM-GT3 (FPG >11.6 mmol/L)
- **2) BMI**
  - i) IGT-OB1 and T2DM-OB1 (BMI <27.7kg/m2),
  - ii) IGT-OB2 and T2DM-OB2 (BMI ≥27.7 to 31.9kg/m2), and
  - iii) IGT-OB3 and T2DM-OB3 (BMI >31.9kg/m2)

## Results

Demographic data for the respective glycaemic sub-groups are displayed in Table 1. Participants in each sub-group were of similar age; however, those with glucose intolerance were heavier (weight, BMI) with increased triglycerides and lower HDL cholesterol than those with NGT.

**Table 1:** Participant characteristics

	NGT	IGT	T2DM
N (M/F)	104 (49/55)	85 (46/39)	554 (419/135
Age (years)	59.0 (13.75)	60.0 (15.00)	55.0 (14.00)
Weight (kg)	73.1 ± 14.59	86.7 ± 15.74^***^	87.5 ± 16.85^***^
Height (m)	1.67 (0.11)	1.67 (0.14)	1.70 (0.13)^*, †^
BMI (kg/m^2^)	25.2 (5.12)	30.7 (6.33)^***^	29.7 (6.84)^***^
Total cholesterol (mmol/L)	5.4 (1.30)	5.1 (1.20)	5.3 (1.60)
Triglycerides (mmol/L)	1.2 (1.06)	1.9 (1.02)^**^	2.0 (1.50)^***, †^
LDL cholesterol (mmol/L)	3.3 (1.30)	3.1 (1.17)	3.2 (1.40)
HDL cholesterol (mmol/L)	1.4 (0.40)	1.1 (0.36)^***^	1.1 (0.40)^***^

Adjusted significance key: * < 0.05, ** < 0.01, *** < 0.001 vs. NGT; † < 0.05, †† < 0.01, ††† < 0.001 vs. IGT

### Analysis according to FPG

#### MTT

**Fig. 2:**
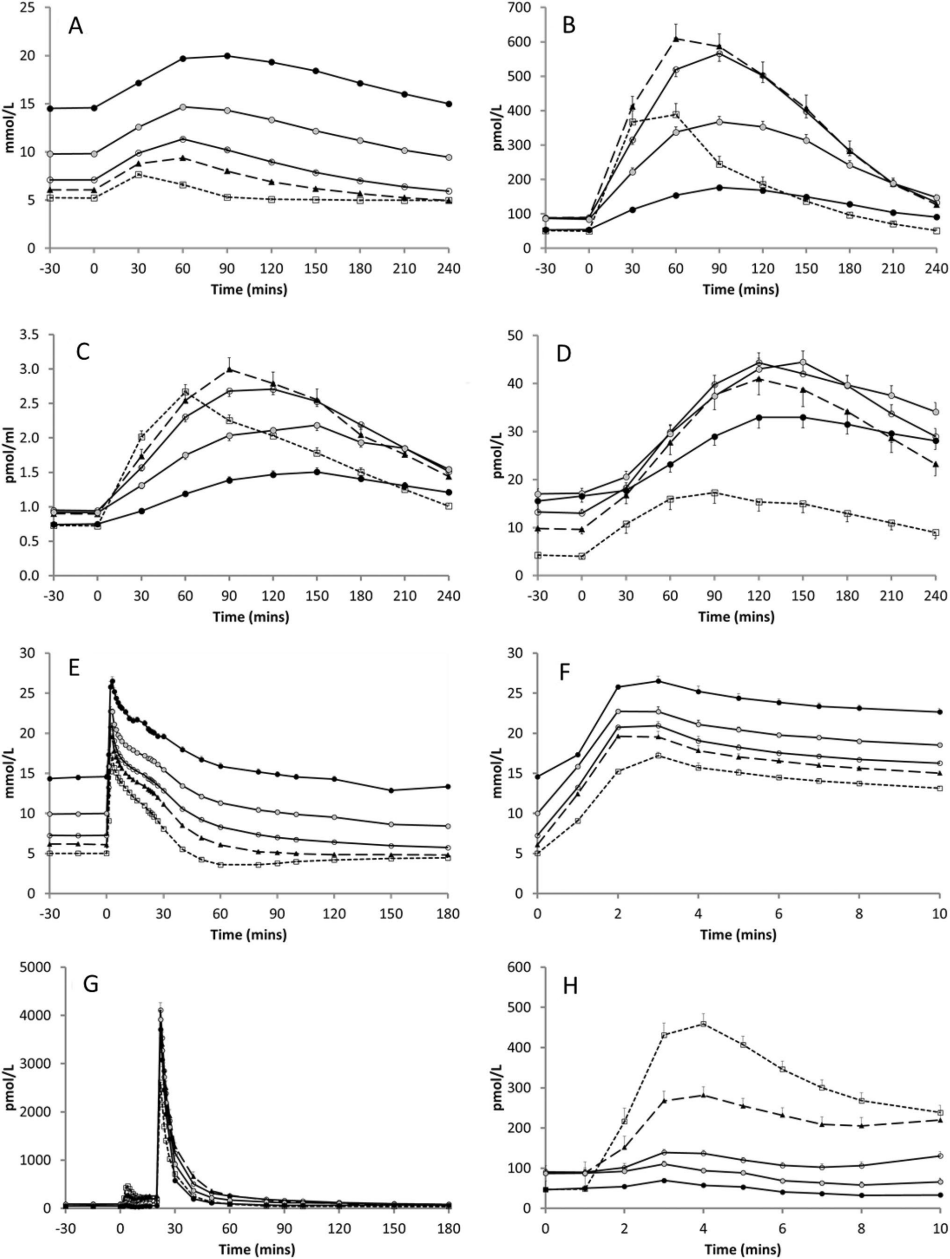
Mean (SEM) plasma **a** glucose, **b** insulin, **c** C-peptide and **d** intact proinsulin responses during the MTT and **e** glucose, **f** glucose (0–10 min post-glucose bolus), **g** insulin and **h** acute insulin response during the FSIVGTT in subjects with NGT, IGT and T2DM stratified by tertiles of FPG. *Dotted line* = *NGT, dashed line* = *IGT, solid line* = *T2DM; open circle* = *T2DM-GT1, grey circle* = *T2DM-GT2, solid circle* = *T2DM-GT3*

The plasma glucose, insulin, C-peptide and proinsulin responses to the MTT are displayed in Fig. 2a–d. In each of the increasing FPG sub-groups, a progressively greater and later post-meal glucose peak was observed (Fig. 2a). Fasting insulin concentrations were higher in the glucose-intolerant sub-groups except those with the highest FPG, whereas peak insulin was only greater in IGT and those T2DM with FPG < 8.5 mmol/L before decreasing with further increasing FPG (Fig. 2b). C-peptide displayed a similar response to insulin (Fig. 2c). In contrast, fasting and peak intact proinsulin was elevated compared to NGT in all glucose-intolerant sub-groups (Fig. 2d). The fasting insulin/glucose ratio was only greater than NGT in the participants with IGT, while no increase in peak insulin/glucose ratio in any of the glucose-intolerant sub-groups was observed (Table 2).

**Table 2:** Measured (a) and derived (b) parameters according to glycaemic sub-group

	NGT	IGT	T2DM-GT1	T2DM-GT2	T2DM-GT3
*(a)*					
N	104	85	180	179	195
Fasting gluc (mmol/L)	5.1 (0.30)	6.1 (0.83)	7.3 (1.10)^***,†††^	9.6 (1.38)^***,†††, ‡‡‡^	14.3 (3.70)^***,†††,‡‡‡,§§§^
C_max_ gluc (mmol/L)	7.9 (1.50)	9.5 (1.65)^*^	11.6 (1.97)^***,†††^	14.9 (2.70)^***,†††, ‡‡‡^	19.7 (4.45)^***,†††,‡‡‡,§§§^
Fasting ins (pmol/L)	39.0 (26.87)	77.0 (59.00)^***^	71.5 (61.75)^***^	67.0 (57.00)^***^	43.0 (42.75)^†††, ‡‡‡^
C_max_ ins (pmol/L)	352 (323.1)	673 (319.0)^***^	563 (491.5)^*^	394 (275.0)^†††, ‡‡‡^	171 (181.0)^***, †††, ‡‡‡, §§§^
Fasting intact PI (pmol/L)	4.0 (2.00)	8.0 (8.95)^**^	11.0 (11.50)^***, †^	15.0 (14.20)^***, †††, ‡^	11.5 (15.58)^***, ††^
C_max_ intact PI (pmol/L)	19.0 (20.00)	44.0 (33.50)^***^	45.0 (36.00)^***^	42.0 (35.75)^***^	29.5 (32.66)^*, ‡‡‡, §§^
Fasting C-pep (pmol/ml)	0.70 (0.37)	0.86 (0.55)^*^	0.91 (0.56)^**^	0.90 (0.63)^*^	0.70 (0.53)^‡‡‡, §^
C_max_ C-pep (pmol/ml)	2.69 (1.21)	3.02 (2.08)	2.92 (1.35)	2.37 (1.41)^*, †††,‡‡‡^	1.66 (1.15)^***, †††, ‡‡‡, §§§^
*(b)*					
Fasting I/G (pmol/mmol)	8.1 (5.43)	12.6 (10.43)^*^	11.1 (9.89)	8.1 (5.85)^†††, ‡‡^	3.2 (3.43)^***, †††, ‡‡‡, §§§^
Max I/G (pmol/mmol)	53.5 (42.34)	67.3 (35.06)	50.1 (39.04)^†^	26.8 (19.35)^***,†††, ‡‡‡^	9.5 (9.39)^***, †††, ‡‡‡, §§§^
Acute insulin response (pmol/L)	410 (289.8)	244.2 (217.3)^**^	145 (103.0)^***, †††^	87 (95.5)^***, †††, ‡^	52 (56.3)^***, †††, ‡‡‡, §§^
Beta-cell function (M_1_)	76.9 (54.31)	48.7^**^	30.0^***, ††^	16.3^***, †††, ‡‡‡^	9.4 (9.99)^***, †††, ‡‡‡, §§§^
Disposition index (DI)	276.3 (325.0)	55.0 (74.7)^***^	30.0 (40.9)^***^	14.5 (16.8)^***, †††^	6.8 (11.2)^***, †††, ‡‡‡^
Insulin sensitivity (SI)	3.89 (3.343)	1.47 (1.605)^***^	0.93 (1.093)^***^	0.71 (0.914)^***,^^†^	0.66 (0.904)^***,^^†^

Adjusted significance key: * < 0.05, ** < 0.01, *** < 0.001 vs. NGT; † < 0.05, †† < 0.01, ††† < 0.001 vs. IGT; ‡ < 0.05, ‡‡ < 0.01, ‡‡‡ < 0.001 vs. T2DM-GT1, § < 0.05, §§ < 0.01, §§§ < 0.001 vs. T2DM-GT2

#### FSIVGTT

Responses to the FSIVGTT are displayed in Fig. 2e–h. Following intravenous glucose administration, plasma glucose rapidly increased, reaching a progressively higher peak with increasing FPG (Fig. 2e). Immediately following administration of the intravenous glucose, a large acute insulin response was observed in participants with NGT, peaking within 4 min (Fig. 2g–h). The response in the glucose-intolerant sub-groups became progressively stunted with increasing FPG, falling to negligible levels in those with FPG > 8.5 mmol/L (Fig. 2h, Table 2).

### Analysis according to BMI

#### MTT

The plasma glucose, insulin, C-peptide and proinsulin responses to the MTT are displayed in Supplementary Fig. 1(A–D). Increasing BMI was not associated with any change in either fasting or peak glucose in subjects with IGT; however, in those with T2DM, with BMI ≥ 27.7 kg/m^2^, the fasting and peak glucose was lower than for BMI < 27.7 kg/m^2^. In T2DM, there were a progressive increase in fasting and peak insulin with increasing BMI and a similar increase in the insulin/glucose ratio (Supplementary Table 3a). In both IGT and T2DM, fasting and peak C-peptide and intact proinsulin levels were increased in those with the highest BMI (Supplementary Fig. 1C, 1D).

#### FSIVGTT

Responses to the FSIVGTT are displayed in Supplementary Fig. 1 (E–H). No differences in the glucose peak were observed with increasing BMI in either the IGT or T2DM groups; however, a significant increasing trend in acute insulin response was observed with increasing BMI in both IGT and T2DM (Supplementary Fig. 1H).

### Insulin sensitivity and beta-cell function

#### Analysis according to FPG

**Fig. 3:**
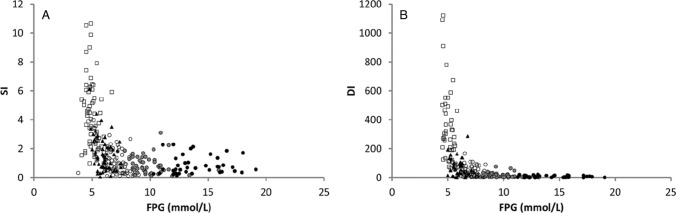
Relationship between fasting glucose and **a** insulin sensitivity and **b** beta-cell function (disposition index). *Open square* = *NGT, solid triangle* = *IGT, open circle* = *T2DM-GT1, grey circle* = *T2DM-GT2, solid circle* = *T2DM-GT3*

Insulin sensitivity and beta-cell function calculated during the FSIVGTT and MTT are displayed in Fig. 3a and b, respectively. Insulin sensitivity fell in a hyperbolic fashion with increasing FPG and was significantly reduced in IGT compared to NGT, decreasing further in T2DM; however, only a small additional deterioration was observed as FPG increased above 7 mmol/L (Fig. 3a). Subjects with IGT displayed a reduction in insulin sensitivity of approximately 60% of that observed in those with NGT, with a further reduction to approximately 20% in T2DM (Table 2). Beta-cell function as measured by disposition index dramatically fell in the early period of glucose intolerance (Fig. 3b) with IGT displaying only approximately 20% that of NGT and continued to fall by half in each increasing glucose-intolerant FPG sub-group (Table 2).

#### Analysis according to BMI

Insulin sensitivity and beta-cell function calculated during the FSIVGTT and MTT are displayed in Supplementary Fig. 2A and 2B, respectively. In both IGT and T2DM, SI was only reduced in those with the highest BMI. Disposition index did not change significantly with increasing BMI in IGT; however, a significant reduction was observed in T2DM with the greatest BMI (Supplementary Table 3b).

## Discussion

The two predominant defects in the pathogenesis of glucose intolerance are beta-cell dysfunction and reduced insulin sensitivity. The reported influence of these defects, however, can depend on multiple factors including the stage of development of glucose intolerance, assay methodology and challenge tests employed and the clinical circumstances of the individual under investigation. Consequently, making direct comparisons between studies is limited.

We present here an assessment of the glycaemic and hormonal responses to two different carbohydrate challenge tests, with derived indices to represent both beta-cell function and insulin sensitivity, applied across a spectrum of participants with NGT, IGT and newly diagnosed, treatment naïve T2DM, collected over a period of over 20 years. The assay antibodies employed for determination of the hormonal parameters were highly sensitive and specific, without the high cross-reactivities observed in earlier assays used in many previous studies. By stratifying the participants according to glycaemic status, the changes in beta-cell function and insulin sensitivity responses to both carbohydrate challenge tests could be viewed in the face of deteriorating glucose control.

In response to the meal, with worsening glycaemia, the postprandial glucose levels were seen to reach progressively increased and delayed peaks. In parallel, insulin concentrations followed the well-recognised ‘Starling’s curve of the pancreas’ pattern [30], where an initial increase in FPG in those with IGT and T2DM was associated with an increase in the insulin levels before falling towards, and eventually below normal levels despite the continued rise in FPG. The postprandial insulin levels began to diminish at a lower FPG than that observed for fasting insulin. When viewed in relation to the ambient glucose level, the postprandial insulin response was never greater than that seen in NGT in glucose intolerance, with a deficient and continually worsening response with increasing FPG in T2DM.

Unlike insulin, the fasting and postprandial proinsulin remained significantly elevated compared to NGT in all of the glucose-intolerant sub-groups. This difference, added to the high cross-reactivity with proinsulin in early and less specific insulin immunoassays, may partly explain discrepancies in the literature with respect to changes in ‘insulin’ response with worsening glycaemia. In addition, the continued elevation of proinsulin in all of the glucose-intolerant sub-groups may offer the potential for its use as a biomarker of glucose intolerance.

Following administration of the glucose bolus during the FSIVGTT, all of the glucose-intolerant participants displayed a severely blunted acute insulin response that continued to decrease to almost non-existent levels as FPG continued to increase in T2DM.

A modelled index of beta-cell function (postprandial beta-cell responsiveness) was estimated for all subjects. By utilising peripheral C-peptide rather than insulin concentrations during the MTT in the model, the pre-hepatic beta-cell response was determined. A hyperbolic decrease in beta-cell function was observed between NGT and IGT and subsequently between IGT and T2DM, continuing to fall significantly in each increasing T2DM FPG sub-group. Insulin sensitivity, derived from the FSIVGTT, also fell in a hyperbolic manner, falling rapidly at lower FPG levels, reaching a nadir in T2DM, all the significant changes occurring during the transition from NGT to IGT and subsequently to T2DM. In contrast to beta-cell function, no further significant changes were observed with increasing FPG in the participants with T2DM.

Our findings that the decrease in beta-cell function is relentless with increasing FPG, whereas the decrease in insulin sensitivity is predominantly in the early stages of glucose intolerance (IGT), reaching a nadir on the advent of T2DM corroborate those observations from previous studies [4, 31] that show beta-cell dysfunction to be a constant contributor in the spectrum of glucose intolerance.

Beta-cell function and insulin sensitivity displayed a hyperbolic relationship. In NGT, a decreased beta-cell function was associated with an increase in insulin sensitivity and vice versa. In those with IGT, a similar relationship was observed; however, the relationship was shifted downwards towards the origin, with a further shift in those with T2DM. This is in agreement with previous oral glucose and euglycaemic clamp studies which noted the relationship [32, 33].

The disposition index is considered the ‘gold-standard’ method of assessing beta-cell function as it ‘corrects’ for the prevailing insulin sensitivity. While displaying a similar overall trend to the more independent measure of beta-cell function, the magnitude of fall of disposition index, however, was far greater at lower FPG, falling by approximately 80% in IGT, in agreement with previous findings of an 80–85% loss of beta-cell function in the upper tertile of IGT [4]. This highlights that when taking into account the prevailing state of the insulin sensitivity, the beta-cell dysfunction is more severe than would be detected using an independent measure.

The main limitation of this study is its cross-sectional nature and long duration. Further limitations include the minimal number of individuals with isolated IFG in the analysis and also the difference in observed BMI between the NGT and glucose-intolerant groups, thus identifying the impact of increasing obesity on both insulin sensitivity and insulin secretion across the entire glycaemic continuum is difficult. The study does, however, have strengths including the large cohort of study participants, with a broad spectrum of glucose tolerance. Those, who were IGT and T2DM, were all newly diagnosed, had received minimal lifestyle advice and were all treatment naïve at the time of investigation. Participants undergoing the meal tolerance test all consumed the same standardised mixed meal, while those who had the intravenous glucose tolerance test all underwent the insulin-modified protocol, allowing more successful modelling of the insulin sensitivity. Those that underwent both carbohydrate challenge tests did so within a few days, ensuring that the derived indices of insulin sensitivity and beta-cell function were performed in the same participants under similar conditions, and also allowed calculation of the disposition index.

In summary, in this large cohort of untreated participants spanning the spectrum of glucose tolerance, increased glycaemia was associated with decreased insulin sensitivity and beta-cell function. The changes in insulin sensitivity were predominant in the early stages, i.e. at the lower levels of FPG observed between NGT and IGT, whereas beta-cell function continued to decrease with increasing glycaemia, and would therefore determine the progression of the disease. We feel these findings present a definitive view of the contribution of both decreased insulin sensitivity and beta-cell dysfunction to the development of glucose intolerance, established as they are in participants who were newly diagnosed, not influenced by lifestyle or pharmacological intervention and determined using well-characterised specific methodologies. The findings also continue to endorse the overall traditionally held views of the pathophysiology of glucose intolerance.

## Supplementary Information

Below is the link to the electronic supplementary material.

Supplementary file1 (PPTX 340 KB)

Supplementary file2 (PPTX 88 KB)

Supplementary file3 (DOCX 28 KB)
