# Supplementary figures and images for: The pathophysiology of glucose intolerance in newly diagnosed, untreated T2DM
Source: Acta Diabetol. 2021 Sep 24;59(2):207–15. doi: 10.1007/s00592-021-01785-9 (PMC8841334; doi:10.1007/s00592-021-01785-9)

## Slide 1
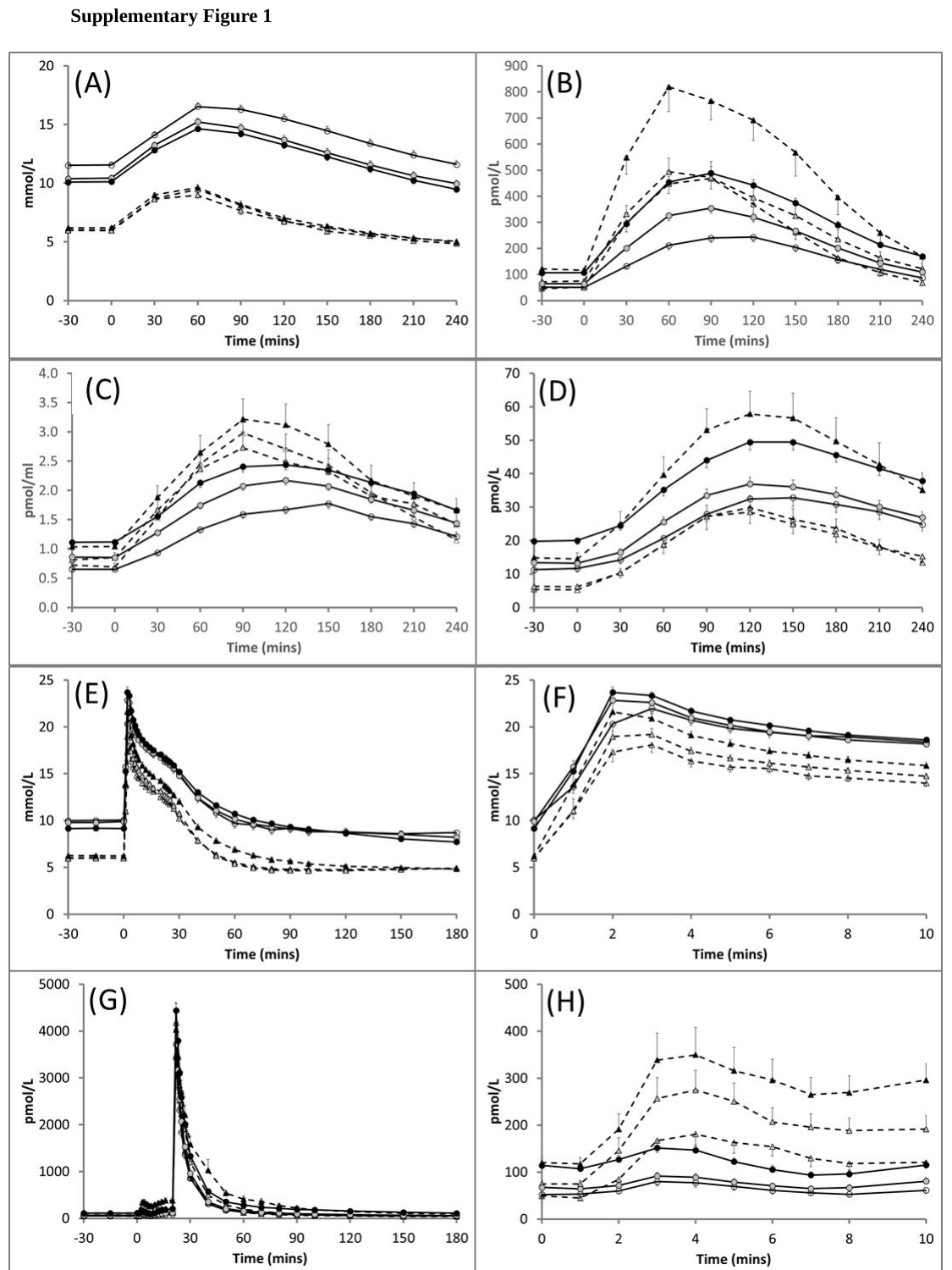

Supplementary Figure 1
Adjusted significance Key:

Supplement: Supplementary file 1 — Supplementary file1 (PPTX 340 KB) [file 592_2021_1785_MOESM1_ESM.pptx]

## Slide 1
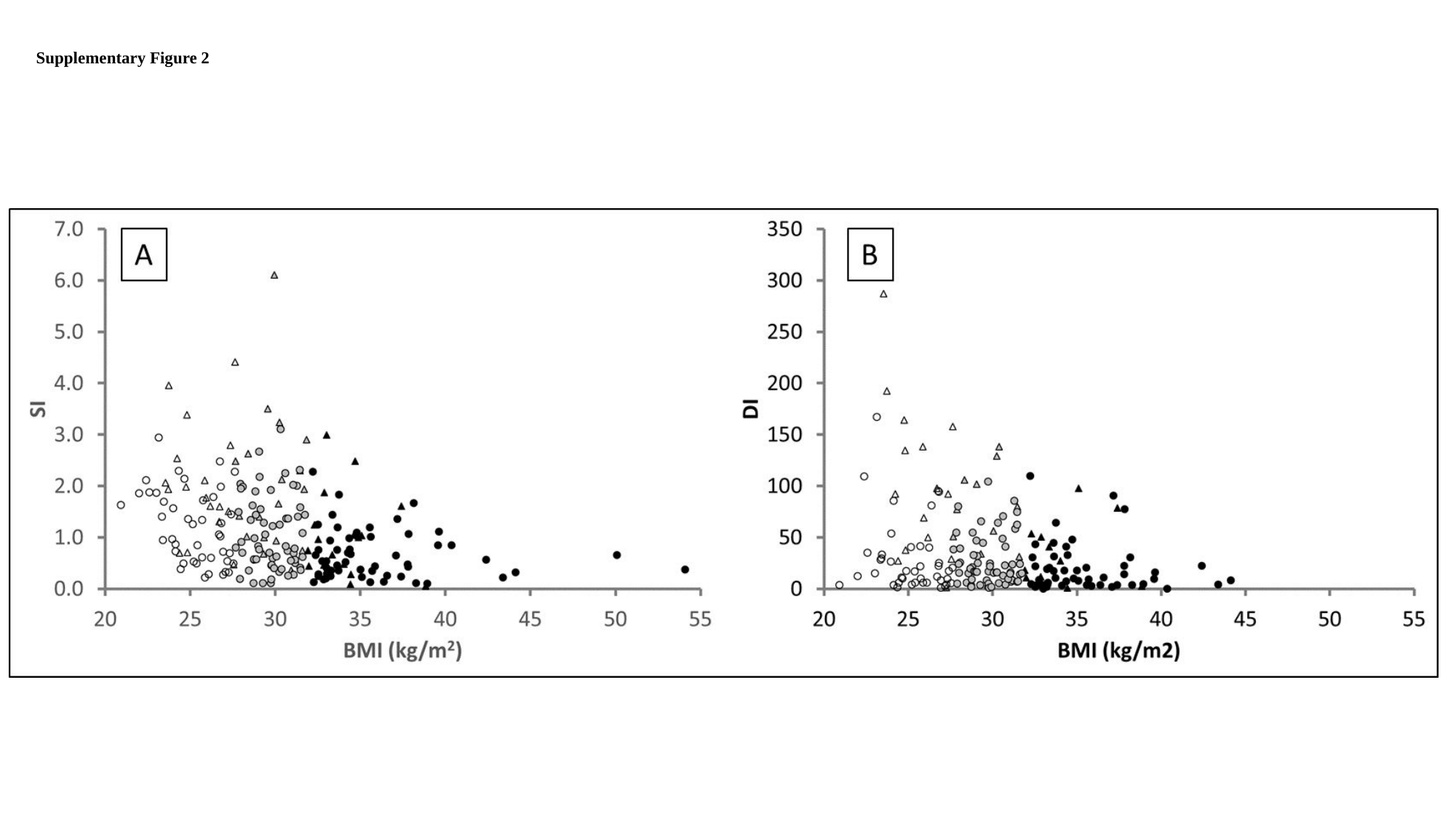

Supplementary Figure 2
Adjusted significance Key:

Supplement: Supplementary file 2 — Supplementary file2 (PPTX 88 KB) [file 592_2021_1785_MOESM2_ESM.pptx]
