# Supplementary material for: The pathophysiology of glucose intolerance in newly diagnosed, untreated T2DM
Source: Acta Diabetol. 2021 Sep 24;59(2):207–15. doi: 10.1007/s00592-021-01785-9 (PMC8841334; doi:10.1007/s00592-021-01785-9)
Supplement: Supplementary file 3 — Supplementary file3 (DOCX 28 KB) [file 592_2021_1785_MOESM3_ESM.docx]

**Supplementary Table 1** Composition of the 500 kcal Mixed Meal Tolerance Test

| 15gram Wheat biscuit cereal |
| --- |
| 10gram Skimmed milk powder (reconstituted) |
| 250ml Pineapple juice |
| Chicken sandwich comprising: 60gram wholemeal bread, 10gram polyunsaturated margarine, 50gram white meat chicken |

**Supplementary Table 2** Sampling timepoints for (a) 500 kcal MTT and (b) FSIVGTT

|  | **Time of sampling (minutes)** |
| --- | --- |
| **(a) MTT** | -30, 0*, 30, 60, 90, 120, 150, 180, 210, 240 |
|  | * = Start of meal. Consumed within 10 minutes |
| **(b) FSIVGTT** | -30, -15, 0^‡^, 1, 2, 3, 4, 5, 6, 7, 8, 10, 12, 14, 16, 20^‡‡^, 22, 23, 24, 25, 27, 30, 40, 50, 60, 70, 80, 90, 100, 120, 180 |
|  | ^‡^ = 50% dextrose bolus administered intravenously over 2 minutes  ^‡‡^ = Insulin bolus (Actrapid) administered intravenously |

**Supplementary Table 3** Measured (a) and derived (b) parameters according to BMI sub-group

(a)

|  | **IGT-OT1** | **IGT-OT2** | **IGT-OT3** | **T2DM-OT1** | **T2DM-OT2** | **T2DM-OT3** |
| --- | --- | --- | --- | --- | --- | --- |
| N | 31 | 23 | 31 | 186 | 185 | 184 |
| Fasting gluc (mmol/L) | 6.0 (0.70) | 6.0 (0.90) | 6.3 (0.80) | 11.2 (6.32) | 9.7 (5.30) | 9.4 (4.00) ^‡^ |
| C_max_ gluc (mmol/L) | 9.8 (1.50) | 9.2 (1.20) | 9.6 (1.70) | 16.7 (6.70) | 14.8 (8.80) ^‡^ | 14.5 (4.88) ^‡‡^ |
| Fasting ins (pmol/L) | 51.0 (46.00) | 75.0 (50.24) | 04.0 (86.68) ^**^ | 40.0 (33.85) | 57.0 (52.25) ^‡‡^ | 95.0 (74.50) ^‡‡‡, §§§^ |
| C_max_ ins  (pmol/L) | 591 (294.6) | 540 (348.1) | 844 (616.5) | 222 (270.8) | 348 (343.0) ^‡‡^ | 485 (442.0) ^‡‡‡, §§^ |
| Fasting C-pep (pmol/ml) | 0.78 (0.51) | 0.85 (0.38) | 0.97(0.59) | 0.58 (0.40) | 0.82 (0.51) ^‡‡‡^ | 1.03 (0.60) ^‡‡‡, §§§^ |
| C_max_ C-pep (pmol/ml) | 3.07 (1.68) | 2.70 (1.92) | 3.02 (2.99) | 1.73 (1.44) | 2.35 (1.42) ^‡‡^ | 2.65 (1.55) ^‡‡‡, §^ |
| Fasting intact PI (pmol/L) | 5.0 (5.50) | 7.0 (4.83) | 14.0 (10.00) ^**, ††^ | 10.0 (9.00) | 10.0 (9.00) | 16.5 (16.87) ^‡‡‡, §§§^ |
| C_max_ intact PI (pmol/L) | 28.0 (31.75) | 30.5 (22.00) | 54.0 (43.50) ^*, †^ | 26.0 (32.00) | 32.0 (30.50) | 49.0 (37.00) ^‡‡‡, §§§^ |

^Adjusted significance Key:
*<0.05, **<0.01, ***<0.001 vs. IGT-OT1; †<0.05, ††<0.01, †††<0.001 vs. IGT-OT2;
‡<0.05, ‡‡<0.01,‡‡‡<0.001 vs. T2DM-OT1, §<0.05, §§<0.01, §§§<0.001 vs. T2DM-OT2^

(b)

|  | **IGT-OT1** | **IGT-OT2** | **IGT-OT3** | **T2DM-OT1** | **T2DM-OT2** | **T2DM-OT3** |
| --- | --- | --- | --- | --- | --- | --- |
| Fasting I:G (pmol/mmol) | 8.8 (7.75) | 12.9 (8.99) | 165 (14.06) ^**^ | 3.8 (4.11) | 6.1 (6.73) ^‡‡‡^ | 10.3 (9.34) ^‡‡‡, §§§^ |
| Max I:G (pmol/mmol) | 61.9 (26.18) | 58.2 (40.18) | 80.1 (64.49) ^**,^ ^†^ | 14.7 (20.96) | 25.0 (29.50) ^‡‡‡^ | 35.2 (40.02) ^‡‡‡, §§§^ |
| Acute insulin response (pmol/L) | 211 (192.0) | 254 (212.0) | 309 (241.2) ^*^ | 53 (71.8) | 84 (78.5) | 142 (120.5) ^‡‡‡, §§§^ |
| Beta-cell function  (M_1_) | 45.0 (28.87) | 50.3 (29.46) | 47.8 (37.07) | 12.8 (16.18) | 17.6 (18.68) ^‡‡^ | 17.4 (25.60) ^‡‡^ |
| Disposition Index (DI) | 80.6 (114.73) | 78.6 (78.43) | 27.3 (42.86) | 13.9 (30.21) | 16.7 (31.85) | 9.0 (18.65) § |
| Insulin sensitivity  (SI) | 1.86 (1.65) | 1.54 (1.81) | 0.85 (1.07) ^*^ | 1.05 (1.22) | 0.84 (1.04) | 0.53 (0.69) ^‡‡, §^ |

^Adjusted significance Key:
*<0.05, **<0.01, ***<0.001 vs. IGT-OT1; †<0.05, ††<0.01, †††<0.001 vs. IGT-OT2;
‡<0.05, ‡‡<0.01,‡‡‡<0.001 vs. T2DM-OT1, §<0.05, §§<0.01, §§§<0.001 vs. T2DM-OT2^
